# Dabigatran and Argatroban Diametrically Modulate Thrombin Exosite Function

**DOI:** 10.1371/journal.pone.0157471

**Published:** 2016-06-15

**Authors:** Calvin H. Yeh, Alan R. Stafford, Beverly A. Leslie, James C. Fredenburgh, Jeffrey I. Weitz

**Affiliations:** Departments of Medicine and Biochemistry and Biomedical Sciences, McMaster University and the Thrombosis and Atherosclerosis Research Institute, Hamilton, Ontario, Canada; University Hospital Medical Centre, GERMANY

## Abstract

Thrombin is a highly plastic molecule whose activity and specificity are regulated by exosites 1 and 2, positively-charged domains that flank the active site. Exosite binding by substrates and cofactors regulates thrombin activity by localizing thrombin, guiding substrates, and by inducing allosteric changes at the active site. Although inter-exosite and exosite-to-active-site allostery have been demonstrated, the impact of active site ligation on exosite function has not been examined. To address this gap, we used surface plasmon resonance to determine the effects of dabigatran and argatroban, active site-directed inhibitors, on thrombin binding to immobilized γ_A_/γ_A_-fibrin or glycoprotein Ibα peptide via exosite 1 and 2, respectively, and thrombin binding to γ_A_/γ′-fibrin or factor Va, which is mediated by both exosites. Whereas dabigatran attenuated binding, argatroban increased thrombin binding to γ_A_/γ_A_- and γ_A_/γ′-fibrin and to factor Va. The results with immobilized fibrin were confirmed by examining the binding of radiolabeled thrombin to fibrin clots. Thus, dabigatran modestly accelerated the dissociation of thrombin from γ_A_/γ_A_-fibrin clots, whereas argatroban attenuated dissociation. Dabigatran had no effect on thrombin binding to glycoprotein Ibα peptide, whereas argatroban promoted binding. These findings not only highlight functional effects of thrombin allostery, but also suggest that individual active site-directed thrombin inhibitors uniquely modulate exosite function, thereby identifying potential novel mechanisms of action.

## Introduction

The substrate specificity of thrombin is dependent on exosites 1 and 2, positively-charged domains that flank the active site [1,2]. These exosites modulate the reactivity of thrombin by providing initial binding sites for substrates, inhibitors or cofactors, sterically hindering other interactions, and by allosterically modifying the active site. Exosite 1 is more versatile because it (a) serves as a docking site for substrates, (b) redirects thrombin activity by binding thrombomodulin, and (c) mediates thrombin inhibition by hirudin and heparin cofactor II. In contrast, exosite 2 has a more limited role because it mainly serves to tether or localize thrombin. For example, exosite 2 contributes to thrombin binding to platelet glycoprotein (Gp) Ibα [3,4] and, by binding heparin, accelerates the inhibition of thrombin by the heparin-antithrombin complex. Exosite 2 also binds the γ′-chain of fibrinogen, thereby mediating a bivalent, high affinity interaction with the variant γ_A_/γ′-fibrinogen [5]. In contrast, because the γ_A_-chain lacks a thrombin binding site, thrombin binds the bulk γ_A_/γ_A_-fibrinogen with lower affinity solely via exosite 1 [6,7]. Both exosites of thrombin also are involved in the interaction of thrombin with factor V, and activated factor V (factor Va) retains affinity for thrombin [8–10].

Numerous studies have shown that thrombin is subject to allosteric modulation. For example, binding of Na^+^ to a conserved site on thrombin increases the catalytic activity of thrombin by altering the conformation of the active site [11,12]. Likewise, ligand binding to exosites 1 or 2 can induce allosteric changes at the active site and/or the reciprocal exosite. Thus, binding of the hirudin peptide [13–15], platelet PAR-1 peptide [13,16], or thrombomodulin [17,18] to exosite 1 or GpIbα [19] or prothrombin fragment 2 [14] to exosite 2 elicits conformational changes at the active site. Likewise, binding of prothrombin fragment 2, γ′-peptide (an analog of the thrombin-binding domain on the γ′-chain of fibrinogen), or HD22 (an exosite 2-binding aptamer) attenuates exosite 1-mediated thrombin interactions with fibrin and other ligands, thereby providing evidence of inter-exosite allostery [20–24]. Previous studies have demonstrated that the exosite to active site connection is bidirectional, such that ligand binding to the active site of thrombin induces reciprocal allosteric changes at the exosites [21,22]. However, the studies were performed with peptide ligands, and it remains unknown whether the same bidirectional effects occur with native ligands or in functional assays. To address this gap, we examined the effects of dabigatran, argatroban, and dansylarginine N-(3-ethyl-1,5-pentanediyl)amide (DAPA), active site-directed small molecules that inhibit thrombin with Ki values of 4.5 nM [25], 19 nM [26], and 45 nM [27], respectively, on thrombin binding to immobilized γ_A_/γ_A_-fibrin, γ_A_/γ′-fibrin, factor Va, and GpIbα peptide. In addition, the effects of these inhibitors on the binding of radiolabeled thrombin to fibrin clots and its subsequent dissociation were examined.

## Experimental Procedures

### Materials

#### Reagents

Human thrombin and plasminogen-free fibrinogen were from Enzyme Research Laboratories (South Bend, IN). Fibrinogen was immunodepleted of factor XIII as described [28]. Human factors Va and XIII and DAPA were from Haematologic Technologies Inc. (Essex Junction, VT). Recombinant thrombin with Arg residues 93, 97, and 101 changed to Ala (RA-thrombin) was a generous gift from Dr. C. Esmon (Oklahoma Medical Research Foundation). Prionex was from Pentapharm (Basel, Switzerland). γ_A_/γ_A_- and γ_A_/γ′-fibrinogen were isolated and characterized as previously described [7,28,29]. Batroxobin from the venom of *Bathrops atrox moojeni* was from Pentapharm (Basel, Switzerland). D-Phe-Pro-Arg (FPR) chloromethyl ketone was from Calbiochem (EMD Millipore, Toronto ON). Chromozym-Thrombin (Chz-Th) was from Hyphen BioMed (Neuville sur Oise, France). Active dabigatran was generously provided by Dr. J. van Ryn (Boehringer-Ingelheim, Biberach, Germany), whereas argatroban was a gift from Dr. D. Stump (Genentech, South San Francisco, CA). Recombinant hirudin was from Dade-Behring (Marburg, Germany). Chloramine T and sodium metabisulfite were from Sigma-Aldrich and Bolton-Hunter reagent was from Pierce Biotechnology (Rockford, IL). The synthetic peptide corresponding to the thrombin exosite 2-specific binding site at residues 269–286 on GpIbα, (Asp-Glu-Gly-Asp-Thr-Asp-Leu-Tyr(PO3)-Asp-Tyr(PO3)-Tyr(PO3)-Pro-Glu-Glu-Asp-Thr-Glu-Gly; GpIbα269-286ppp) was synthesized with three phosphorylated Tyr residues by Mimotopes (Minneapolis, MN) [30,31].

#### Radiolabeled thrombin

To 10 μl of 0.2 M sodium borate, pH 8.0, and 10 μl (1 mCi) of Na^125^I (McMaster University Nuclear Reactor, Hamilton, ON) was added 5 μl of 1.5 mM Bolton-Hunter reagent in DMSO. The reaction was initiated by adding 10 μl of 5 mg/ml chloramine T in 20 mM sodium phosphate, 150 mM NaCl, pH 7.4 (PBS). After 1 min at 23°C, the reaction was stopped by addition of 10 μl of 12 mg/ml sodium metabisulfite in PBS. To the reaction mixture was added 150 μg of thrombin and after incubation for 1 hr at 23°C, the reaction was terminated by addition of 100 μl of 0.2 M glycine in 0.2 M sodium borate, pH 8.0. Labeled thrombin was isolated on a PD-10 column (GE Healthcare, Baie d’Urfe, PQ), and eluted with 20 mM Tris-HCl, 150 mM NaCl, pH 7.4 (TBS) containing 0.01% Tween-80 (TBS-Tw). The specific activity of the labeled thrombin was 3.8 x 10^8^ cpm/mg.

#### Active site-blocked thrombin

Thrombin (3–3.5 mg/ml) was incubated with 2-fold molar excess of FPR chloromethyl ketone at 23°C. After 30 min, absence of residual activity of 2 μM FPR-thrombin was determined at 405 nm with 200 μM Chz-Th in a Spectramax plate reader (Molecular Devices, Sunnyvale CA). The sample was washed with TBS and concentrated using an Amicon Ultra 4 ml– 3000 MW centrifugal cartridge (Millipore) [14].

### Methods

#### SPR

Studies were performed using a Biacore T200 (GE Healthcare). γ_A_/γ_A_-fibrinogen, γ_A_/γ′-fibrinogen, and factor Va were immobilized to separate flow cells of a CM5 sensor chip using the amine coupling kit from GE Healthcare [32]. Briefly, after injecting the 1-ethyl-3-(3-dimethylaminopropyl)-carbodiimide hydrochloride/N-hydroxysuccinimide mixture into the flow cell at a rate of 10 μl/min for 420 s, 50 μg/ml of γ_A_/γ_A_- or γ_A_/γ′-fibrinogen or 28 μg/ml factor Va in 10 mM acetate buffer, pH 4.5, was injected at a rate of 5 μl/min until about 8000 response units (RU) were immobilized. High levels of protein were adsorbed because previous studies indicate that only 30% of bound fibrinogen is in an accessible orientation, and because of the large difference in molecular weight between the bound ligands and the analyte thrombin [28]. Flows cells were then washed with 1 M ethanolamine for 420 s, followed by the running buffer composed of 20 mM HEPES, 150 mM NaCl, pH 7.4 (HBS) containing 0.01% Tween-80 (HBS-Tw) and 5 mM CaCl_2_. To convert the immobilized fibrinogen to fibrin, flow cells were subjected to successive 60-min injections of up to 1 μM thrombin [24,28,32]. Quantitative conversion to fibrin was evidenced by saturable decline in RU following thrombin treatment [33].

To measure binding to immobilized γ_A_/γ_A_-fibrin, γ_A_/γ′-fibrin or factor Va, increasing concentrations of up to 5 μM thrombin or FPR-thrombin were injected into the flow cells. To measure the effects of dabigatran, argatroban, and DAPA on thrombin binding to fibrin or factor Va, 250–500 nM thrombin or FPR-thrombin was incubated with 0–5 μM inhibitor and injected into the flow cells. Because of the fast on- and off-rates, all SPR experiments were performed at a flow rate of 25 μl/min [28]. Association times were 120 s and dissociation was monitored by washing the flow cells with HBS-Tw for 600 s. Between runs, flow cells were regenerated with a 45 s wash with HBS-Tw containing 0.5 M CaCl_2_.

The K_d_ values for one-site binding of thrombin to γ_A_/γ_A_-fibrin were determined from plots of RU values at equilibrium (Req) *versus* thrombin or FPR-thrombin concentration using Biacore T200 Evaluation software v 1.0. The rapid approach to equilibrium at the low flow rate nullified any potential mass transport concerns. Nonspecific binding was accounted for by subtracting the control RU values obtained from the unmodified flow cell. To calculate two-site binding of thrombin to γ_A_/γ′-fibrin or factor Va, plots were subjected to kinetic analysis using a two-site binding model. EC_50_ values for the effect of dabigatran, argatroban, or DAPA on thrombin binding were determined by plotting Req values against the concentration of active site inhibitor and using non-linear regression to fit these to a rectangular hyperbola.

To examine the effect of the active site inhibitors on exosite 2-mediated binding, 1 mg of GpIbα269-286ppp in HBS was biotinylated at its NH_2_-terminus by incubation with a 10-fold molar excess of Hook-Sulfo-NHS-LC biotin (G-Biosciences, St. Louis, MO) for 1 h at 23°C. After separation from unincorporated reagent by chromatography on Sephadex G15, fractions containing biotinylated peptide were identified by monitoring absorbance at 204 nm. Streptavidin (Sigma) was attached to flow cells of a CM5 chip to about 12000 RU and biotin (b)-GpIbα269-286ppp was then adsorbed to about 150 RU above the streptavidin background. Aliquots of thrombin or RA-thrombin up to 4 μM in HBS-Tw were injected into the flow cell at 25 μl/min for 80 s, followed by HBS-Tw for 200 s. Between runs, flow cells were regenerated with 1 M NaCl for 30 s. To measure the effect of dabigatran, argatroban, or DAPA on this interaction, 250 nM thrombin was injected in the presence of 0–5000 nM dabigatran, argatroban, or DAPA. EC50 values were determined as above. To quantify the effects of inhibitors on binding affinity, 0–2 μM thrombin was injected in the presence of 2.5 μM active site inhibitor at 25 μl/min for 60 s. Binding affinities were calculated as described above.

#### Effect of dabigatran, argatroban and DAPA on ^125^I-thrombin binding to fibrin clots

To a series of 1.5-ml micro-centrifuge tubes containing 0–1 μM dabigatran or argatroban, 0–4 μM DAPA, or 0–0.1 μM hirudin were added 10 nM ^125^I-thrombin, 3 μM γ_A_/γ_A_-fibrinogen or 1 μM γ_A_/γ′-fibrinogen, 2 mM CaCl_2_ and 1 unit/mL batroxobin in TBS-Tw in a total volume of 100 μl [24,28,32]. Batroxobin was used to induce clotting because its activity is unaffected by the inhibitors (not shown). After incubation for 1 h at 23°C, clots were pelleted by centrifugation at 14,000 × g for 4 min and 50-μl aliquots of supernatant were removed and counted for radioactivity using a gamma counter (Wizard^2^ 2470, Perkin-Elmer) to determine the concentration of unbound ^125^I-thrombin. EC_50_ values for the effect of the inhibitors were calculated by fitting plots of bound ^125^I-thrombin versus the inhibitor concentration by non-linear regression analysis to an equation for a rectangular hyperbola. K_i_ values were then determined using the Cheng-Prusoff equation and the dissociation constant of ^125^I-thrombin for fibrinogen as determined in a separate experiment.

#### Dissociation of ^125^I-thrombin from fibrin clots

Dissociation of ^125^I-thrombin from preformed fibrin clots was performed as described [28]. Briefly, clots were formed around plastic inoculation loops by clotting a 130 μl solution containing 3 μM γ_A_/γ_A_-fibrinogen and 30 nM factor XIII in TBS-Tw containing 2 mM CaCl_2_ with 10 nM ^125^I-thrombin. After incubation for 1 h, clots were immersed in 50 ml plastic tubes containing 10 ml of 100 nM dabigatran, argatroban or DAPA, or 2 M NaCl in TBS-Tw at 23°C. At defined intervals, aliquots were removed, counted for radioactivity, and returned to the tubes. Plots of residual radioactivity versus time were analyzed using a 2-phase exponential decay equation [28].

*Statistical analysis*–All experiments were performed at least three times. Results are presented as the mean ± standard deviation (SD). Paired data were compared using Student t-tests; whereas group means were compared using one-way ANOVA followed by Tukey's test for multiple comparisons. For all analyses, p values less than 0.05 were considered statistically significant.

## Results

### SPR analysis of the interaction of thrombin with immobilized fibrin or factor Va

**Fig 1:**
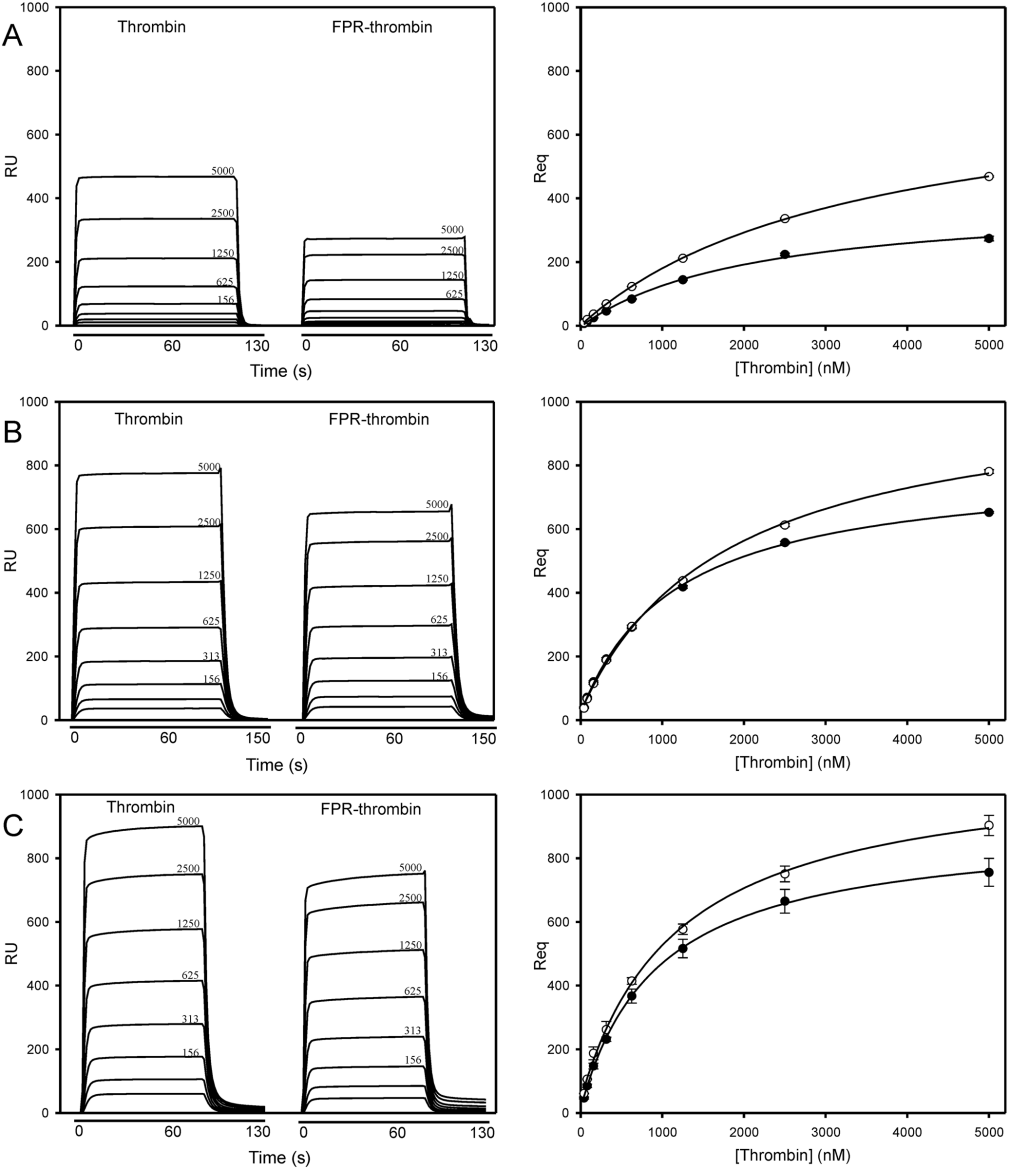
Binding of thrombin or FPR-thrombin to immobilized γ_A_/γ_A_-fibrin, γ_A_/γ′-fibrin, or factor Va. Increasing concentrations of thrombin or FPR-thrombin up to 5000 nM were injected into individual flow cells of a CM5 biosensor chip containing immobilized (A) γ_A_/γ_A_-fibrin, (B) γ_A_/γ′-fibrin, or (C) factor Va. Samples were injected for 120 s at 25 μl/min, followed by a 600 s wash to monitor dissociation. *Left panels*; sensorgram tracings of representative runs for thrombin and FPR-thrombin are shown in succession. Analyte concentrations are 39, 78, 156, 313, 625, 1250, 2500, 5000 nM, or as indicated. *Right panels*; from each sensorgram, the amount of thrombin (open circles) or FPR-thrombin (closed circles) bound at equilibrium after background correction (Req) was determined and plotted against the thrombin concentration. Data points represent the mean ± S.D. of 3 experiments, and the *lines* represent nonlinear regression analysis.

Increasing concentrations of thrombin or FPR-thrombin were injected into flow cells containing immobilized γ_A_/γ_A_-fibrin, γ_A_/γ′-fibrin, or factor Va [24,28]. Because sensorgram profiles rapidly reached equilibrium, Req values were determined and plotted versus the analyte concentration to determine K_d_ (Fig 1). Non-specific binding is not a major concern because prothrombin exhibited only weak affinity for immobilized fibrin, and thrombin did not bind to immobilized ovalbumin (not shown). As expected, more thrombin and FPR-thrombin bound to γ_A_/γ′-fibrin than to γ_A_/γ_A_-fibrin. Binding to γ_A_/γ_A_-fibrin was via a single lower affinity site, whereas binding to γ_A_/γ′-fibrin occurred through higher and lower affinity interactions, demonstrating involvement of both exosites (Table 1) [28,32]. Affinities are similar to those reported previously using SPR and fibrin clots [7,24]. More thrombin and FPR-thrombin also bound to factor Va than to γ_A_/γ_A_-fibrin. The higher affinities for factor Va than for γ_A_/γ_A_-fibrin are consistent with interaction mediated by both exosites [8–10]. The affinities of FPR-thrombin for fibrin and factor Va were ~50% higher than those of active thrombin (p < 0.001).

**Table 1:** Dissociation constants (K_d_) for the binding of thrombin to fibrin, factor Va and GpIbα269-286ppp. The binding of thrombin or FPR-thrombin to immobilized γ_A_/γ_A_-fibrin, γ_A_/γ′-fibrin, factor Va or GpIbα269-286ppp was quantified using SPR. K_d_ values for thrombin binding to γ_A_/γ_A_-fibrin and GpIbα269-286ppp were determined by steady state analysis, whereas bivalent binding of thrombin to γ_A_/γ′-fibrin and factor Va was determined by kinetic analysis for a two-site model using BIAevaluation software. K_d_ values are shown as mean ± S.D. for three separate experiments.

	Thrombin		FPR-thrombin	
	K_d1_	K_d2_	K_d1_	K_d2_
Target	(nM)			
**γ**_**A**_**/γ**_**A**_**-fibrin**	3887 ± 36	-	2110 ± 140	-
**γ**_A_/**γ′-fibrin**	2631 ± 18	211.6 ± 0.9	1434.2 ± 56.8	110.4 ± 14.0
**Factor Va**	1471 ± 73	219.8 ± 189.4	831.2 ± 13.7	130.4 ± 17.7
**GpIbα269-286ppp**	306 ± 11	-	211 ± 1	-

### Effects of dabigatran, argatroban or DAPA on thrombin binding to immobilized fibrin or factor Va

**Fig 2:**
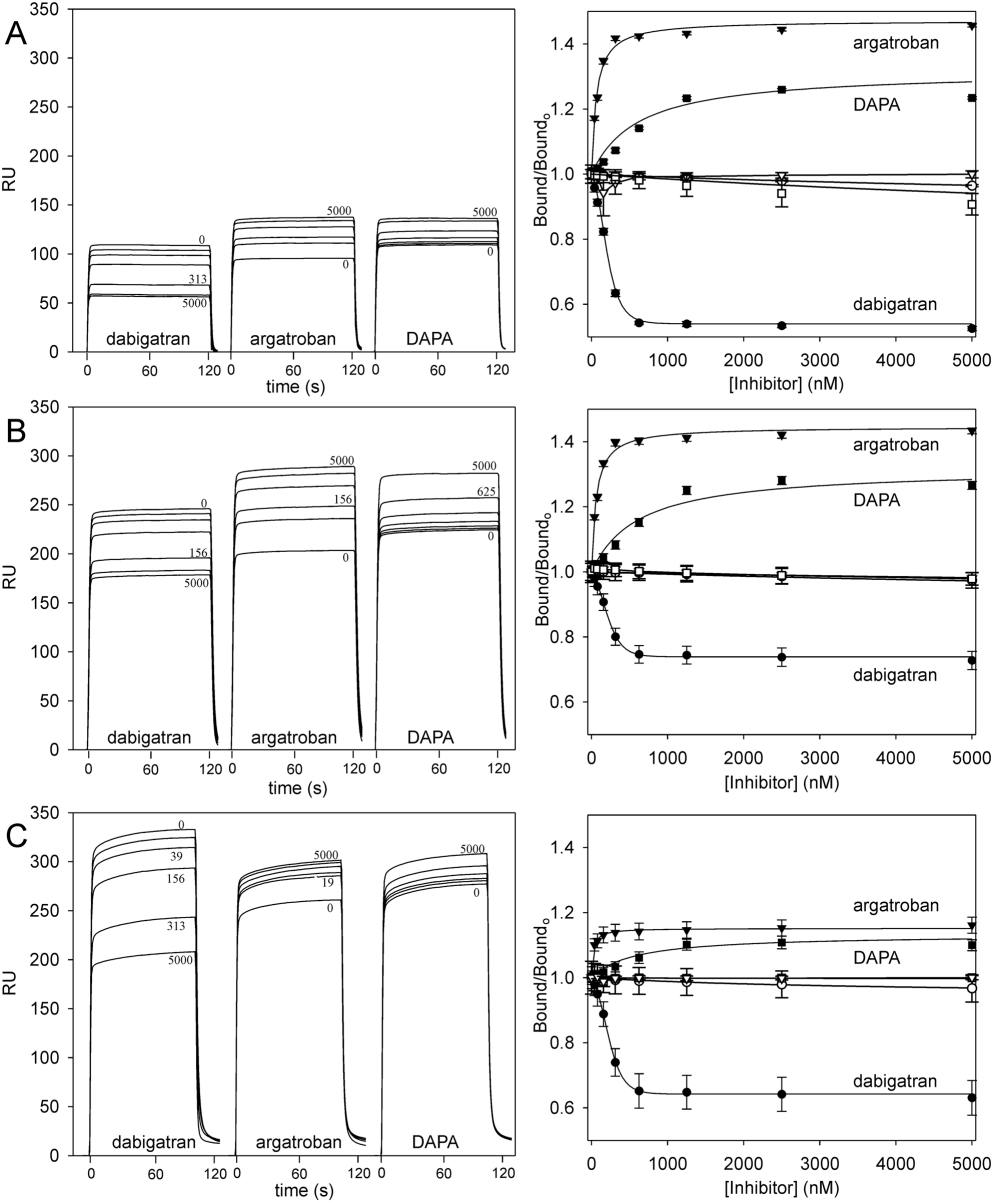
Effect of dabigatran, argatroban, or DAPA on thrombin binding to immobilized γ_A_/γ_A_-fibrin, γ_A_/γ′-fibrin, or factor Va. Increasing concentrations of dabigatran (closed circles), argatroban (closed triangles), or DAPA (closed squares) up to 5 μM were pre-incubated with thrombin prior to injection into individual flow cells containing immobilized (A) γ_A_/γ_A_-fibrin, (B) γ_A_/γ′-fibrin, or (C) factor Va. Sample injection was followed by a 600 s wash with buffer to monitor dissociation. *Left panels*; representative sensorgrams of the effects of increasing concentrations of dabigatran, argatroban or DAPA on thrombin binding are shown in succession. *Right panels*; the ratios of Req values of thrombin bound in the absence (Bound_o_) and presence (Bound) of dabigatran, argatroban or DAPA are plotted against the input inhibitor concentrations. Open symbols represent the effects of the inhibitors on FPR-thrombin binding to γ_A_/γ_A_-fibrin, γ_A_/γ′-fibrin, or factor Va. Data points represent the mean ± S.D. of 3 experiments, and the *lines* represent nonlinear regression analysis.

Binding of 250–500 nM thrombin or FPR-thrombin to immobilized γ_A_/γ_A_-fibrin, γ_A_/γ′-fibrin, or factor Va was assessed by SPR in the presence of increasing concentrations of dabigatran, argatroban, or DAPA. Dabigatran reduced thrombin bound to γ_A_/γ_A_-fibrin, γ_A_/γ′-fibrin, and factor Va in a concentration-dependent manner (Fig 2) with EC_50_ values of 184.6 ± 4.3 nM, 182.4 ± 15.0 nM, and 204.2 ± 17.0 nM, respectively. At saturation, dabigatran reduced thrombin binding to γ_A_/γ_A_-fibrin, γ_A_/γ′-fibrin, and factor Va by 47.6 ± 0.4%, 28.4 ± 1.6%, and 37.9 ± 3.2%, respectively (Table 2). As a control, experiments using FPR-thrombin in place of thrombin demonstrated no effect of dabigatran on binding, confirming that dabigatran binds specifically to the active site (Fig 2).

**Table 2:** Effect of dabigatran, argatroban, or DAPA on thrombin binding to γ_A_/γ_A_-fibrin, γ_A_/γ′-fibrin, factor Va, or GpIbα269-286ppp. The effect of 0–5000 nM dabigatran, argatroban, or DAPA on the binding of thrombin to immobilized γ_A_/γ_A_-fibrin, γ_A_/γ′-fibrin, factor Va or GpIbα269-286ppp was quantified using SPR. EC_50_ values were determined by plotting the Req values against the inhibitor concentration and fitting by nonlinear regression analysis using BIAevaluation software. Change is the calculated difference between thrombin binding in the absence of inhibitors and that determined at saturating inhibitor concentration. Values are shown as mean ± S.D. for three separate experiments.

	Dabigatran		Argatroban		DAPA	
Target	EC_50_ (nM)	Change (%)	EC_50_ (nM)	Change (%)	EC_50_ (nM)	Change (%)
**γ**_**A**_**/γ**_**A**_**-fibrin**	184.6 ± 4.3	- 47.6 ± 0.4	62.4 ± 4.8	+ 47.2 ± 1.0	514.1 ± 24.0	+ 25.1 ± 0.5
**γ**_**A**_**/γ′-fibrin**	182.4 ± 15.0	- 28.4 ± 1.6	59.4 ± 5.1	+ 44.5 ± 2.3	515.9 ± 31.0	+ 27.7 ± 1.5
**Factor Va**	204.2 ± 17.0	- 37.9 ± 3.2	23.4 ± 8.8	+ 15.1 ± 3.9	565.1 ± 200	+ 13.2 ± 3.2
**GpIbα269-286ppp**	—	0	161.0 ± 2.6	+17.3 ± 0.6	1288.3 ± 32.3	+25.4 ± 0.0

In contrast to the results with dabigatran, argatroban and DAPA increased thrombin binding to γ_A_/γ_A_-fibrin, γ_A_/γ′-fibrin, and factor Va in a concentration-dependent manner with EC_50_ values for argatroban of 62.4 ± 4.8 nM, 59.4 ± 5.1 nM, and 23.4 ± 8.8 nM, respectively, and for DAPA of 514.1 ± 24.0 nM, 515.9 ± 31.0 nM, and 565.1 ± 200 nM, respectively (Fig 2). Although argatroban and DAPA are reported to bind thrombin with similar affinities, the EC_50_ values for argatroban were more than 10-fold lower than those for DAPA. As observed with dabigatran, neither argatroban nor DAPA had any effect on FPR-thrombin binding to γ_A_/γ_A_-fibrin, γ_A_/γ′-fibrin, or factor Va (Fig 2). Therefore, reversible engagement of the active site by small molecules allosterically modulates the exosite-mediated interactions of thrombin with fibrin and factor Va.

### Effects of dabigatran, argatroban and DAPA on thrombin binding to GpIbα269-286ppp

**Fig 3:**
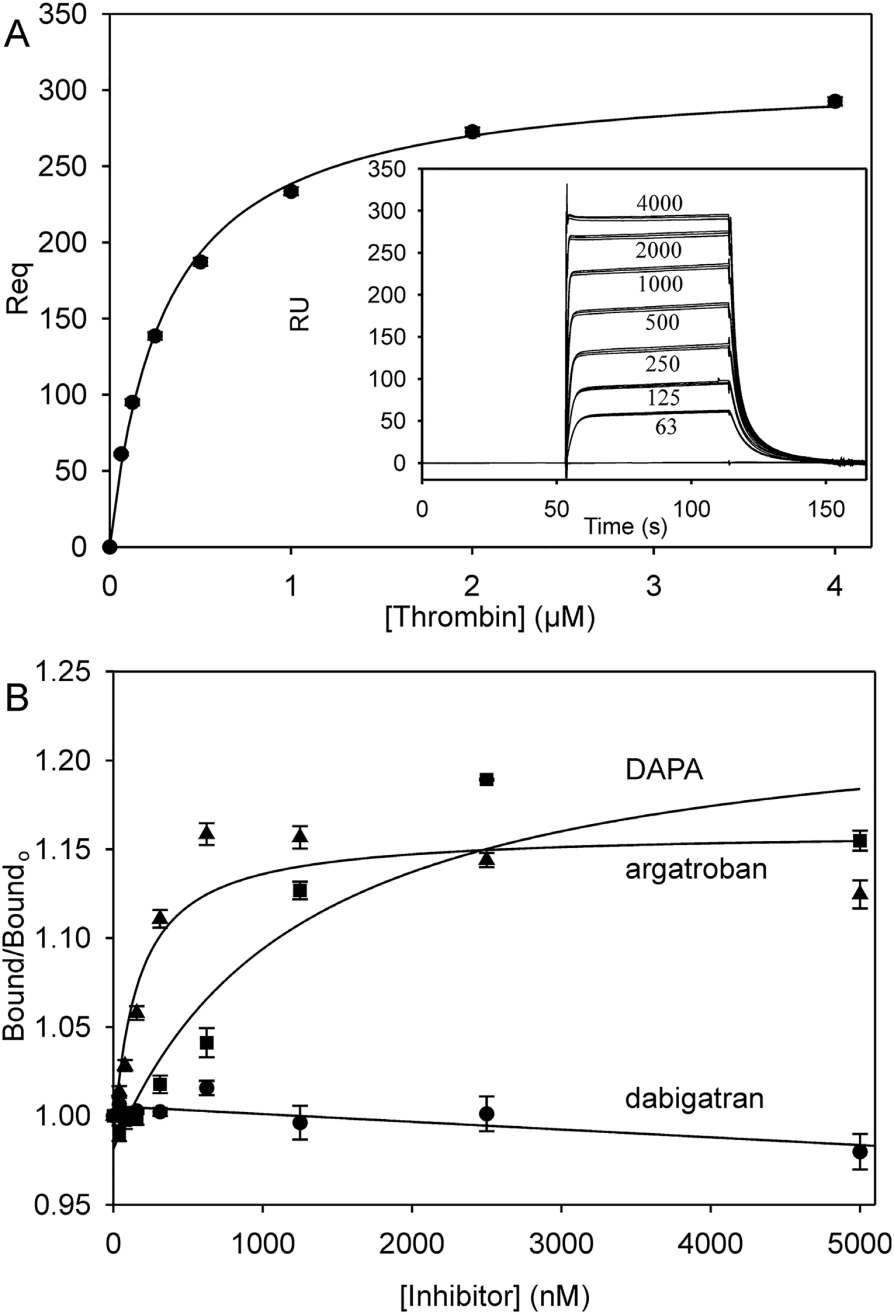
Binding of thrombin to immobilized GpIbα269-286ppp. *A*. Increasing concentrations of thrombin up 4 μM were injected at a rate of 25 μl/min into flow cells of a streptavidin-coated CM5 biosensor chip containing adsorbed biotinlylated GpIbα269-286ppp. Response units (RU) are plotted versus time for the indicated thrombin concentrations (in nM) in triplicate (inset). RU values at equilibrium (Req) were determined and plotted versus the input thrombin concentration. The data were analyzed by nonlinear regression (line) to a rectangular hyperbola to determine K_d_. Symbols represent mean ± SD for from 3 runs. *B*. Thrombin (250 nM) was injected into flow cells containing immobilized biotinlylated GpIbα269-286ppp in the presence of 0–5000 nM dabigatran (circles), argatroban (triangles), or DAPA (squares). Req values were determined, converted to a fraction of that measured in the absence of inhibitor (Bound/Bound_0_), and plotted versus the inhibitor concentration. Data points indicate mean ± SD for 3 determinations, and the *lines* represent nonlinear regression analyses.

Thrombin bound to the immobilized GpIbα269-286ppp peptide with a K_d_ of 306 ± 11 nM (Fig 3A; Table 1); an affinity intermediate between that reported for a similar peptide and for glycocalicin [30,34]. In contrast, RA-thrombin, an exosite 2 variant, did not bind (not shown), consistent with the reported specificity of this interaction for exosite 2 [30]. Thrombin binding to the GpIbα269-286ppp peptide was then quantified in the presence of increasing concentrations of inhibitors. Whereas dabigatran had no effect on thrombin binding, argatroban and DAPA increased the amount of thrombin bound by 17% and 25%, respectively (Fig 3B; Table 2). Binding to GpIbα269-286ppp was then quantified in the presence of 2.5 μM dabigatran, argatroban or DAPA. Whereas thrombin bound with similar affinity in the presence of dabigatran (K_d_ 306 ± 11 nM; not shown) as in its absence, the affinity of thrombin for GpIbα269-286ppp significantly (p < 0.001 by ANOVA) increased by 25–35% in the presence of argatroban or DAPA (K_d_ values of 229 ± 7 nM and 197 ± 5 nM, respectively). These findings demonstrate that occupation of the active site by some, but not all, small molecules increases the affinity of ligands for exosite 2, similar to the observation for exosite 1-dependent interactions.

### Effects of dabigatran, argatroban and DAPA on ^125^I-thrombin binding to fibrin clots

Although the affinities of thrombin and FPR-thrombin for γ_A_/γ_A_- and γ_A_/γ′-fibrin determined by SPR are comparable with those obtained with fibrin clots [28], it was important to confirm that the allosteric response observed using SPR also occurred in a functional assay with three-dimensional fibrin clots. ^125^I-thrombin bound γ_A_/γ_A_- and γ_A_/γ′-fibrin clots with K_d_ values of 3.6 ± 0.3 μM and 1.2 ± 0.2 μM (not shown), respectively; values comparable with those obtained using SPR and with those published previously [32]. Up to 70% of the ^125^I-thrombin bound to fibrin, demonstrating that labeling had little effect on the capacity of thrombin to bind to fibrin.

**Fig 4:**
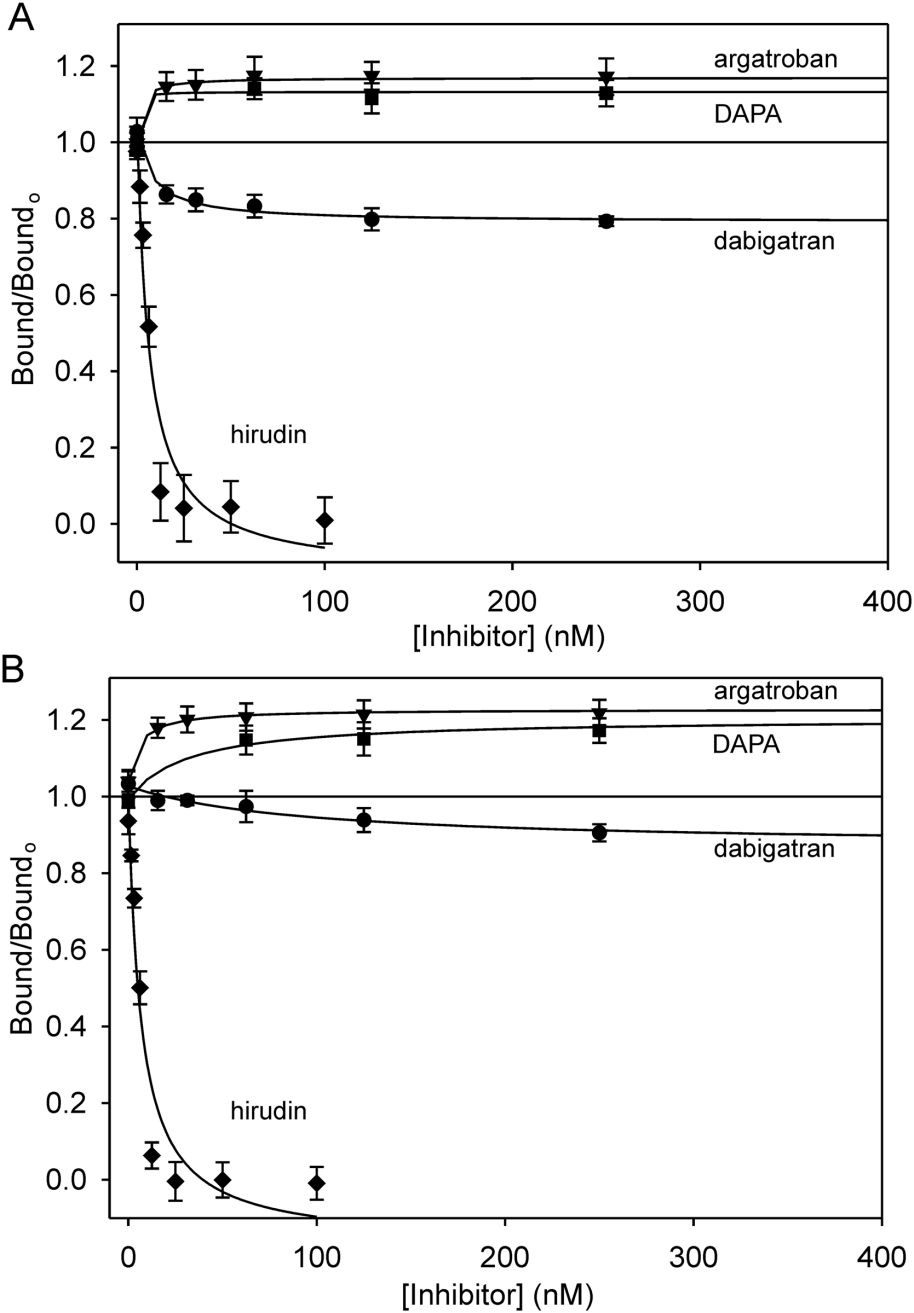
Effect of dabigatran, argatroban, DAPA, or hirudin on the binding of ^125^I-thrombin to fibrin clots. Increasing concentrations of dabigatran (circles), argatroban (triangles), DAPA (squares), or hirudin (diamonds) were added to samples containing 10 nM ^125^I-thrombin, 2 mM CaCl_2_, 1 U/mL batroxobin and (A) 3 μM γ_A_/γ_A_-fibrinogen, or (B) 1 μM γ_A_/γ′-fibrinogen. After incubation for 1 h, clots were pelleted and aliquots of supernatant were counted for radioactivity. The ratios of ^125^I-thrombin bound in the absence or presence of inhibitor are plotted against the inhibitor concentrations. Data points represent the mean ± S.D. of 2–3 experiments, each performed in duplicate, and the *lines* represent nonlinear regression analysis.

Dabigatran reduced ^125^I-thrombin binding to γ_A_/γ_A_- and γ_A_/γ′-fibrin clots in a concentration-dependent manner with K_i_ values of 3.8 ± 1.5 nM and 26.0 ± 4.0 nM, respectively (Fig 4). The K_i_ value for the thrombin interaction with γ_A_/γ_A_-fibrin is comparable with the value of 4.5 nM obtained by chromogenic assay [35]. The K_i_ value is higher with γ_A_/γ′-fibrin than γ_A_/γ_A_-fibrin, likely reflecting the higher affinity, bivalent interaction mediated by both exosites [7]. As observed using SPR, dabigatran produced a 2-fold greater reduction in thrombin binding to γ_A_/γ_A_-fibrin than to γ_A_/γ′-fibrin (p < 0.05); a difference that likely reflects the higher affinity of thrombin for γ_A_/γ′-fibrin. To verify the role of exosite 1 in binding, the effect of hirudin also was examined. As expected, hirudin fully inhibited ^125^I-thrombin binding to γ_A_/γ_A_- and γ_A_/γ′-fibrin. In agreement with the SPR observations, argatroban and DAPA enhanced the binding of ^125^I-thrombin to γ_A_/γ_A_- and γ_A_/γ′-fibrin clots to a similar extent (~15–25%) (Fig 4, Table 3). Collectively, therefore, the data obtained with fibrin clots support the SPR results and suggest that active site engagement induces allosteric changes in the thrombin exosites, with the direction and magnitude determined by the ligand.

**Table 3:** Effect of dabigatran, argatroban, DAPA or hirudin on ^125^I-thrombin binding to fibrin clots. Binding of ^125^I-thrombin to γ_A_/γ_A_- or γ_A_/γ′-fibrin clots in the absence or presence of dabigatran, argatroban, DAPA, or hirudin was determined by measuring the amount of ^125^I-thrombin in the supernatants of compacted clots prepared by incubating γ_A_/γ_A_- or γ_A_/γ′-fibrinogen with a catalytic amount of batroxobin. EC_50_ values were determined by nonlinear regression analysis and converted to K_i_ values by correction for K_d_ of ^125^I-thrombin for fibrin. Change denotes the maximal change in the percentage of bound ^125^I-thrombin compared with that in the absence of inhibitor.

	Dabigatran		Argatroban		DAPA		Hirudin	
Ligand	K_i_ (nM)	Change (%)	K_i_ (nM)	Change (%)	K_i_ (nM)	Change (%)	K_i_ (nM)	Change (%)
**γ**_**A**_**/γ**_**A-**_**fibrin**	3.8 ± 1.5	-21.4 ± 3.3	2.4 ± 1.9	+18.3 ± 5.0	2.1 ± 0.9	+17.0 ± 2.7	3.4 ± 0.3	-100
**γ**_**A**_**/γ′-fibrin**	26.0 ± 4.0	-9.4 ± 1.4	7.0 ± 5.6	+26.1 ± 0.4	19.6 ± 19.1	+20.1 ± 3.6	3.4 ± 0.1	-100

### Effects of dabigatran, argatroban and DAPA on ^125^I-thrombin dissociation from fibrin clots

**Fig 5:**
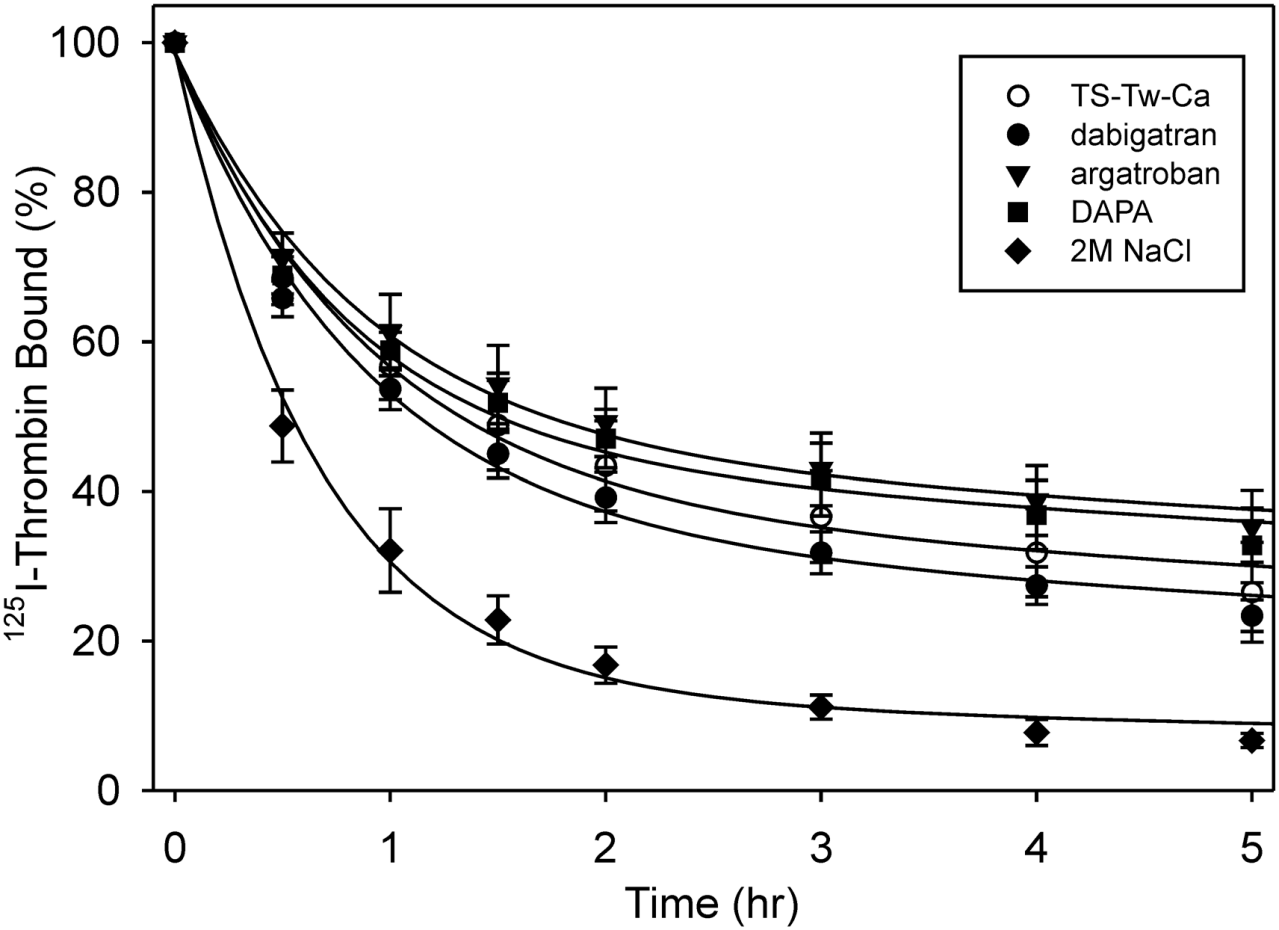
Effect of dabigatran, argatroban, DAPA, or hirudin on the dissociation of ^125^I-thrombin from fibrin clots. Clots were formed around plastic loops by incubating 3 μM γ_A_/γ_A_-fibrinogen and 30 nM factor XIII with 10 nM ^125^I-thrombin in the presence of 2 mM CaCl_2_ for 1 h. Clots were immersed in 10 ml TSTwCa buffer alone, or buffer containing 100 nM dabigatran, argatroban, or DAPA, or 2 M NaCl. At intervals, aliquots were removed and counted for radioactivity. Residual counts were normalized with respect to time zero and plotted versus time. Data were analyzed by two phase exponential decay (lines) to determine the half-lives of dissociation. Symbols represent the mean ± SD of 4 experiments.

To determine whether the alteration in the affinity of thrombin for fibrin in the presence of active site inhibitors influences the amount of thrombin associated with fibrin, dissociation from fibrin clots was examined [28]. Clots formed from γ_A_/γ_A_-fibrinogen in the presence of ^125^I-thrombin were incubated in buffer in the absence or presence of active site inhibitors and the amount of ^125^I-thrombin released from the clot over time was quantified. Biphasic analysis was used to evaluate the fast non-adsorbent and slow bound phases of dissociation of thrombin from fibrin. In the control, the bound fraction dissociated with a half-life of 20.5 ± 3.1 h, whereas in the presence of 2 M NaCl where ionic interactions are abrogated, the rate of dissociation was significantly faster (p = 0.02) at 14.2 ± 1.2 h (Fig 5). The half-life values in the presence of 2.5 μM dabigatran, argatroban, and DAPA were 17.7 ± 2.3 h, 25.5 ± 2.9 h, and 23.7 ± 2.9 h, respectively. Although these values were not significantly different from the control, there was a trend for more rapid dissociation in the presence of dabigatran. However, the half-lives in the presence of argatroban and DAPA were significantly longer than that in the presence of dabigatran (p = 0.01 and 0.02, respectively). These data are consistent with the effects of the inhibitors on the affinity of thrombin for fibrin, and confirm the differences in response between dabigatran and argatroban or DAPA.

## Discussion

The purpose of this study was to determine whether occupation of the active site of thrombin by small molecules induces functional changes at its exosites. We chose fibrin, factor Va and GpIbα269-286ppp as targets for three reasons. First, their use enabled studies with unmodified, active thrombin. Second, the mode of interaction of thrombin with these ligands has been well characterized; thus, binding of thrombin to γ_A_/γ_A_-fibrin and GpIbα269-286ppp is solely dependent on exosite 1 and exosite 2, respectively, whereas thrombin binding to γ_A_/γ′-fibrin and factor Va requires both exosites. Third, the use of native ligands such as fibrin and factor Va would complement previous results obtained with peptide ligands, and the use of fibrin would enable confirmation in functional assays. Dabigatran and argatroban were chosen because they are highly specific for thrombin and their interactions with the active site of thrombin have been extensively investigated and because both agents are in clinical use. DAPA was chosen to complement the results with argatroban because both incorporate an Arg group that is essential for specificity [36,37].

Dabigatran saturably reduces thrombin binding to γ_A_/γ_A_-fibrin and factor Va by 28–48%, but has no effect on the interaction of thrombin with Gp1bα269-286ppp. Surprisingly, argatroban and DAPA increase thrombin binding to γA/γA-fibrin, γ_A_/γ′-fibrin, factor Va and GpIbα269-286ppp by 13–47%. The observations with fibrin were confirmed using three-dimensional fibrin clots and were not dependent on the affinity of the inhibitors for thrombin because dabigatran, argatroban and DAPA inhibit thrombin with similar nanomolar K_i_ values. DAPA and argatroban also slow the dissociation of thrombin from fibrin clots in a manner consistent with their enhancement of the affinity of thrombin for fibrin. Thus, these studies demonstrate allosteric effects of active site binding on thrombin, and provide evidence that responses are ligand specific.

Investigation into the allosteric regulation of thrombin has shown that binding of a variety of effectors to the exosites modulates the active site. This phenomenon has been attributed to a complex allosteric network within thrombin that is subject to modulation through surface residues [13,16]. Within the active site, residues in the S_1-4_ subsites and the 60- and autolysis-loops contribute to this allosteric network [16,17,38,39]. These regions within the active site make unique contact with individual substrates and are perturbed by exosite ligands, thereby rendering them central to allosteric regulation. It is interesting to compare the interaction of dabigatran, argatroban and DAPA with the active site of thrombin because dabigatran induces effects that differ from those of argatroban and DAPA. Although dabigatran and argatroban engage the S1, S2, and S4 subsites in the active site, they do so in distinct ways [35–37,40]. Thus, the differences in exosite response elicited by the various inhibitors likely reflect unique points of contact within the allosteric network. In an analogous fashion, different exosite 1 ligands evoke unique changes in thrombin activity [14,24,39,41,42], suggesting that the exosites are composed of distinct subdomains [2,43–46]. Consistent with the results observed here with fibrin and factor Va, argatroban has previously been shown to increase the affinity of hirudin peptide for exosite 1 on thrombin [47]. These observations were not limited to exosite 1 because argatroban and DAPA in the current study also increased the affinity of thrombin for GpIbα peptide; an interaction mediated solely by exosite 2. The structural explanations for how different small molecules can bind to active site of thrombin and evoke distinct responses at the exosites remain to be determined.

The observation that binding of dabigatran, argatroban or DAPA to the active site of thrombin induces changes at the exosites is consistent with the hypothesis that changes transmitted over the allosteric network are bidirectional. Most studies have focused on the effect of exosite ligands on active site function. However, calorimetry and fluorescence studies using peptide ligands demonstrate that structural changes that result from ligand binding at the active site or exosite 1 can run in both directions [21,47,48]. This property suggests that covalent occupation of the active site by FPR should alter exosite function; a concept supported by some studies [38,49–52], but not by others [21,53–55]. Results presented here suggest that noncovalent adducts also can modify exosite function, and represent another way by which thrombin can convert between different functional and conformational states [23]. Although extensive investigations of thrombin structure have not provided a unifying model of thrombin allostery, they demonstrate that thrombin is a highly dynamic molecule with numerous conformations and networks of internal communication.

The capacity of dabigatran to attenuate thrombin binding to fibrin and factor Va reveals a unique mechanism by which this inhibitor might function as an anticoagulant. Thus, in addition to inhibiting the catalytic activity of thrombin, dabigatran may displace thrombin from fibrin or platelets. This would reduce the protection of thrombin from inhibition afforded by such binding, and could further attenuate the thrombogenic potential of the thrombus [56,57]. Such displacement would be similar to that produced by exosite-directed ligands, which also displace thrombin from fibrin or platelets [3,58]. Because argatroban promotes thrombin binding to fibrin, it is likely that not all direct thrombin inhibitors benefit from this secondary effect. This raises the possibility that in addition to simple target potency, future efforts at structure-based drug design should consider the secondary effects of inhibitors; effects that may be altered by refining contacts within the active site.

In summary, our results suggest that active site inhibitors modulate thrombin function mediated by the exosites. This reverse direction of allosteric signaling provides greater insight into the dynamic nature of thrombin. Furthermore, different active site-directed agents evoke unique responses, demonstrating that their inhibitory function can be refined. Thus, exploitation of its allosteric network endows thrombin with additional mechanism of regulation without compromising its repertoire of substrates. This demonstrates that the versatility of thrombin is a consequence of the intricate connections between the active site and the exosites.

## References

[1] Krishnaswamy S. The transition of prothrombin to thrombin. J Thromb Haemost. 2013; 11 Suppl 1: 265–276. 10.1111/jth.12217 23809130 PMC3713535

[2] Bock PE, Panizzi P, Verhamme IM. Exosites in the substrate specificity of blood coagulation reactions. J Thromb Haemost. 2007; 5 Suppl 1: 81–94. 17635714 10.1111/j.1538-7836.2007.02496.x PMC2291348

[3] De Candia E, Hall SW, Rutella S, Landolfi R, Andrews RK, De Cristofaro R. Binding of thrombin to glycoprotein Ib accelerates the hydrolysis of Par-1 on intact platelets. J Biol Chem. 2001; 276: 4692–4698. 11084032 10.1074/jbc.M008160200

[4] Zarpellon A, Celikel R, Roberts JR, McClintock RA, Mendolicchio GL, Moore KL et al Binding of alpha-thrombin to surface-anchored platelet glycoprotein Ib(alpha) sulfotyrosines through a two-site mechanism involving exosite I. Proc Nat Acad Sci USA. 2011; 108: 8628–8633. 10.1073/pnas.1017042108 21555542 PMC3102361

[5] Wolfenstein-Todel C, Mosesson MW. Carboxy-terminal amino acid sequence of a human fibrinogen gamma-chain variant (gamma'). Biochemistry. 1981; 20: 6146–6149. 7306501 10.1021/bi00524a036

[6] Meh DA, Siebenlist KR, Mosesson MW. Identification and characterization of the thrombin binding sites on fibrin. J Biol Chem. 1996; 271: 23121–23125. 8798504 10.1074/jbc.271.38.23121

[7] Pospisil CH, Stafford AR, Fredenburgh JC, Weitz JI. Evidence that both exosites on thrombin participate in its high affinity interaction with fibrin. J Biol Chem. 2003; 278: 21584–21591. 12682049 10.1074/jbc.M300545200

[8] Esmon CT, Lollar P. Involvement of thrombin anion-binding exosites 1 and 2 in the activation of factor V and factor VIII. J Biol Chem. 1996; 271: 13882–13887. 8662922 10.1074/jbc.271.23.13882

[9] Segers K, Dahlback B, Bock PE, Tans G, Rosing J, Nicolaes GA. The role of thrombin exosites I and II in the activation of human coagulation factor V. J Biol Chem. 2007; 282: 33915–33924. 17878169 10.1074/jbc.M701123200 PMC2292461

[10] Dharmawardana KR, Olson ST, Bock PE. Role of regulatory exosite I in binding of thrombin to human factor V, factor Va, factor Va subunits, and activation fragments. J Biol Chem. 1999; 274: 18635–18643. 10373475 10.1074/jbc.274.26.18635

[11] Pozzi N, Chen R, Chen Z, Bah A, Di Cera E. Rigidification of the autolysis loop enhances Na(+) binding to thrombin. Biophys Chem. 2011; 159: 6–13. 10.1016/j.bpc.2011.04.003 21536369 PMC3150630

[12] Gohara DW, Di Cera E. Allostery in trypsin-like proteases suggests new therapeutic strategies. Trends Biotechnol. 2011; 29: 577–585. 10.1016/j.tibtech.2011.06.001 21726912 PMC3191250

[13] Lechtenberg BC, Johnson DJ, Freund SM, Huntington JA. NMR resonance assignments of thrombin reveal the conformational and dynamic effects of ligation. Proc Nat Acad Sci USA. 2010; 107: 14087–14092. 10.1073/pnas.1005255107 20660315 PMC2922568

[14] Fredenburgh JC, Stafford AR, Weitz JI. Evidence for allosteric linkage between exosites 1 and 2 of thrombin. J Biol Chem. 1997; 272: 25493–25499. 9325262 10.1074/jbc.272.41.25493

[15] Verhamme IM, Olson ST, Tollefsen DM, Bock PE. Binding of exosite ligands to human thrombin. Re-evaluation of allosteric linkage between thrombin exosites I and II. J Biol Chem. 2002; 277: 6788–6798. 11724802 10.1074/jbc.M110257200

[16] Gandhi PS, Chen Z, Mathews FS, Di Cera E. Structural identification of the pathway of long-range communication in an allosteric enzyme. Proc Nat Acad Sci USA. 2008; 105: 1832–1837. 10.1073/pnas.0710894105 18250335 PMC2538848

[17] Adams TE, Li W, Huntington JA. Molecular basis of thrombomodulin activation of slow thrombin. J Thromb Haemost. 2009; 7: 1688–1695. 10.1111/j.1538-7836.2009.03563.x 19656282 PMC2844540

[18] Gasper PM, Fuglestad B, Komives EA, Markwick PR, McCammon JA. Allosteric networks in thrombin distinguish procoagulant vs. anticoagulant activities. Proc Nat Acad Sci USA. 2012; 109: 21216–21222. 10.1073/pnas.1218414109 23197839 PMC3535651

[19] Li CQ, Vindigni A, Sadler JE, Wardell MR. Platelet glycoprotein Ib alpha binds to thrombin anion-binding exosite II inducing allosteric changes in the activity of thrombin. J Biol Chem. 2001; 276: 6161–6168. 11024046 10.1074/jbc.M004164200

[20] Malovichko MV, Sabo TM, Maurer MC. Ligand binding to anion-binding exosites regulates conformational properties of thrombin. J Biol Chem. 2013; 288: 8667–8678. 10.1074/jbc.M112.410829 23378535 PMC3605685

[21] Treuheit NA, Beach MA, Komives EA. Thermodynamic compensation upon binding to exosite 1 and the active site of thrombin. Biochemistry. 2011; 50: 4590–4596. 10.1021/bi2004069 21526769 PMC3107735

[22] Sabo TM, Farrell DH, Maurer MC. Conformational analysis of gamma' peptide (410–427) interactions with thrombin anion binding exosite II. Biochemistry. 2006; 45: 7434–7445. 16768439 10.1021/bi060360k

[23] Kamath P, Huntington JA, Krishnaswamy S. Ligand binding shuttles thrombin along a continuum of zymogen- and proteinase-like states. J Biol Chem. 2010; 285: 28651–28658. 10.1074/jbc.M110.154914 20639195 PMC2937891

[24] Petrera NS, Stafford AR, Leslie BA, Kretz CA, Fredenburgh JC, Weitz JI. Long range communication between exosites 1 and 2 modulates thrombin function. J Biol Chem. 2009; 284: 25620–25629. 10.1074/jbc.M109.000042 19589779 PMC2757964

[25] Wienen W, Stassen JM, Priepke H, Ries UJ, Hauel N. In-vitro profile and ex-vivo anticoagulant activity of the direct thrombin inhibitor dabigatran and its orally active prodrug, dabigatran etexilate. Thromb Haemost. 2007; 98: 155–162. 17598008

[26] Fitzgerald D, Murphy N. Argatroban: a synthetic thrombin inhibitor of low relative molecular mass. Coron Artery Dis. 1996; 7: 455–458. 8889361

[27] Nesheim ME, Prendergast FG, Mann KG. Interactions of a fluorescent active-site-directed inhibitor of thrombin: Dansylarginine N-(3-Ethyl-1,5-pentanediyl)amide. Biochemistry. 1979; 18: 996–1003. 427102 10.1021/bi00573a010

[28] Fredenburgh JC, Stafford AR, Leslie BA, Weitz JI. Bivalent binding to gamma A/gamma'-fibrin engages both exosites of thrombin and protects it from inhibition by the antithrombin-heparin complex. J Biol Chem. 2008; 283: 2470–2477. 18055456 10.1074/jbc.M707710200

[29] Schaefer AV, Leslie BA, Rischke JA, Stafford AR, Fredenburgh JC, Weitz JI. Incorporation of fragment X into fibrin clots renders them more susceptible to lysis by plasmin. Biochemistry. 2006; 45: 4257–4265. 16566600 10.1021/bi0525730

[30] Lechtenberg BC, Freund SM, Huntington JA. GpIbalpha interacts exclusively with exosite II of thrombin. J Mol Biol. 2014; 426: 881–893. 10.1016/j.jmb.2013.11.027 24316004 PMC3919161

[31] Sabo TM, Maurer MC. Biophysical investigation of GpIbalpha binding to thrombin anion binding exosite II. Biochemistry. 2009; 48: 7110–7122. 10.1021/bi900745b 19591434 PMC2842903

[32] Vu TT, Stafford AR, Leslie BA, Kim PY, Fredenburgh JC, Weitz JI. Batroxobin binds fibrin with higher affinity and promotes clot expansion to a greater extent than thrombin. J Biol Chem. 2013; 288: 16862–16871. 10.1074/jbc.M113.464750 23612970 PMC3675619

[33] Kim PY, Tieu LD, Stafford AR, Fredenburgh JC, Weitz JI. A high affinity interaction of plasminogen with fibrin is not essential for efficient activation by tissue-type plasminogen activator. J Biol Chem. 2012; 287: 4652–4661. 10.1074/jbc.M111.317719 22187433 PMC3281636

[34] De Cristofaro R, De Candia E, Rutella S, Weitz JI. The Asp(272)-Glu(282) region of platelet glycoprotein Ibalpha interacts with the heparin-binding site of alpha-thrombin and protects the enzyme from the heparin-catalyzed inhibition by antithrombin III. J Biol Chem. 2000; 275: 3887–3895. 10660541 10.1074/jbc.275.6.3887

[35] Hauel NH, Nar H, Priepke H, Ries U, Stassen JM, Wienen W. Structure-based design of novel potent nonpeptide thrombin inhibitors. J Med Chem. 2002; 45: 1757–1766. 11960487 10.1021/jm0109513

[36] Brandstetter H, Turk D, Hoeffken HW, Grosse D, Sturzebecher J, Martin PD et al Refined 2.3 A X-ray crystal structure of bovine thrombin complexes formed with the benzmidine and arginine-based thrombin inhibitors NAPAP, 4-TAPAP and MQPA. A starting point for improving antithrombotics. J Mol Biol. 1992; 226: 1085–1099. 1518046 10.1016/0022-2836(92)91054-s

[37] Mathews II, Tulinsky A. Active-site mimetic inhibition of thrombin. Acta Crystallogr D Biol Crystallogr. 1995; 51: 550–559. 15299843 10.1107/S0907444994013132

[38] Croy CH, Koeppe JR, Bergqvist S, Komives EA. Allosteric changes in solvent accessibility observed in thrombin upon active site occupation. Biochemistry. 2004; 43: 5246–5255. 15122890 10.1021/bi0499718

[39] Ng NM, Quinsey NS, Matthews AY, Kaiserman D, Wijeyewickrema LC, Bird et al The effects of exosite occupancy on the substrate specificity of thrombin. Arch Biochem Biophys. 2009; 489: 48–54. 10.1016/j.abb.2009.07.012 19638274

[40] Bode W, Turk D, Karshikov A. The refined 1.9 A X-ray crystal structure of D-Phe-Pro-Arg chloromethylketone-inhibited human a-thrombin: Structure analysis, overall structure, electrostatic properties, detailed active site geometry, and structure-function relationships. Protein Sci. 1992; 1: 426–471. 1304349 10.1002/pro.5560010402 PMC2142221

[41] Ye J, Esmon NL, Esmon CT, Johnson AE. The active site of thrombin is altered upon binding to thrombomodulin. Two distinct structural changes are detected by fluorescence, but only one correlates with protein C activation. J Biol Chem. 1991; 266: 23016–23021. 1660464

[42] Liu LW, Vu TKH, Esmon CT, Coughlin SR. The region of the thrombin receptor resembling hirudin binds to thrombin and alters enzyme specificity. J Biol Chem. 1991; 266: 16977–16980. 1654318

[43] Kretz CA, Stafford AR, Fredenburgh JC, Weitz JI. HD1, a thrombin-directed aptamer, binds exosite 1 on prothrombin with high affinity and inhibits its activation by prothrombinase. J Biol Chem. 2006; 281: 37477–37485. 17046833 10.1074/jbc.M607359200

[44] Abdel Aziz MH, Sidhu PS, Liang A, Kim JY, Mosier PD, Zhou Q et al Designing allosteric regulators of thrombin. Monosulfated benzofuran dimers selectively interact with Arg173 of exosite 2 to induce inhibition. J Med Chem. 2012; 55: 6888–6897. 10.1021/jm300670q 22788964 PMC3416887

[45] Bode W. Structure and interaction modes of thrombin. Blood Cells Mol Dis. 2006; 36: 122–130. 16480903 10.1016/j.bcmd.2005.12.027

[46] Page MJ, MacGillivray RT, Di Cera E. Determinants of specificity in coagulation proteases. J Thromb Haemost. 2005; 3: 2401–2408. 16241939 10.1111/j.1538-7836.2005.01456.x

[47] Parry MAA, Stone SR, Hofsteenge J, Jackman MP. Evidence for common structural changes in thrombin induced by active-site or exosite binding. Biochem J. 1993; 290: 665–670. 8457193 10.1042/bj2900665 PMC1132332

[48] De Cristofaro R, De Candia E, Picozzi M, Landolfi R. Conformational transitions linked to active site ligation in human thrombin: Effect on the interaction with fibrinogen and the cleavable platelet receptor. J Mol Biol. 1995; 245: 447–458. 7837275 10.1006/jmbi.1994.0036

[49] De Cristofaro R, Landolfi R. Thermodynamics of substrates and reversible inhibitors binding to the active site cleft of human α-thrombin. J Mol Biol. 1994; 239: 569–577. 8006969 10.1006/jmbi.1994.1396

[50] Bock PE, Olson ST, Bjork I. Inactivation of thrombin by antithrombin is accompanied by inactivation of regulatory exosite I. J Biol Chem. 1997; 272: 19837–19845. 9242645 10.1074/jbc.272.32.19837

[51] Fredenburgh JC, Stafford AR, Weitz JI. Conformational changes in thrombin when complexed by serpins. J Biol Chem. 2001; 276: 44828–44834. 11584020 10.1074/jbc.M108710200

[52] Li W, Johnson DJ, Adams TE, Pozzi N, De F V, Huntington JA. Thrombin inhibition by serpins disrupts exosite II. J Biol Chem. 2010; 285: 38621–38629. 10.1074/jbc.M110.144964 20889971 PMC2992294

[53] Kroh HK, Tans G, Nicolaes GA, Rosing J, Bock PE. Expression of allosteric linkage between the sodium ion binding site and exosite I of thrombin during prothrombin activation. J Biol Chem. 2007; 282: 16095–16104. 17430903 10.1074/jbc.M610577200 PMC2292469

[54] Figueiredo AC, Clement CC, Zakia S, Gingold J, Philipp M, Pereira PJ. Rational design and characterization of D-Phe-Pro-D-Arg-derived direct thrombin inhibitors. PLoS ONE. 2012; 7: e34354 10.1371/journal.pone.0034354 22457833 PMC3311629

[55] Pozzi N, Acquasaliente L, Frasson R, Cristiani A, Moro S, Banzato A et al beta2 -Glycoprotein I binds to thrombin and selectively inhibits the enzyme procoagulant functions. J Thromb Haemost. 2013; 11: 1093–1102. 10.1111/jth.12238 23578283

[56] Weitz JI, Hudoba M, Massel D, Maraganore J, Hirsh J. Clot-bound thrombin is protected from inhibition by heparin-antithrombin III but is susceptible to inactivation by antithrombin III-independent inhibitors. J Clin Invest. 1990; 86: 385–391. 2384594 10.1172/JCI114723 PMC296739

[57] Becker DL, Fredenburgh JC, Stafford AR, Weitz JI. Exosites 1 and 2 are essential for protection of fibrin-bound thrombin from heparin-catalyzed inhibition by antithrombin and heparin cofactor II. J Biol Chem. 1999; 274: 6226–6233. 10037709 10.1074/jbc.274.10.6226

[58] Naski MC, Fenton JWI, Maraganore JM, Olson ST, Shafer JA. The COOH-terminal domain of hirudin. An exosite-directed competitive inhibitor of the action of alpha-thrombin on fibrinogen. J Biol Chem. 1990; 265: 13484–13489. 2380171

